# Unique chicken B cell development: species-specific mechanisms and contradictory requirements of B cell receptor for post-hatched B cell development

**DOI:** 10.3389/fimmu.2026.1755331

**Published:** 2026-02-05

**Authors:** Seung Je Woo, Thirubasyini Songodan, Jae Yong Han

**Affiliations:** Department of Agricultural Biotechnology and Research Institute of Agriculture and Life Sciences, Seoul National University, Seoul, Republic of Korea

**Keywords:** B cell, bursa of Fabricius, chicken, gene editing, single-cell RNA sequencing

## Abstract

Chicken B cell development represents a remarkable evolutionary divergence from mammalian paradigms, featuring unique three-stage ontogeny centered on the bursa of Fabricius, an avian-specific primary B cell lymphoid organ. Unlike mammals where B cells develop continuously in bone marrow, chickens utilize a temporally restricted program spanning pre-bursal (E5-E14), bursal (E8-hatching), and post-bursal phases (hatching-bursal involution), each characterized by distinct molecular mechanisms and anatomical sites. In this review, we documented chicken B cell development in three developmental phases (pre-bursal to post-bursal phases) and compared it with mammalian B cell development mostly in humans as a representative mammalian model. In chicken, while the embryonic bursa of Fabricius serves as the primary B cell receptor (BCR)-dependent B cell developmental organ, it also supports BCR-independent early colonization followed by extensive activation-induced cytidine deaminase (AID)-mediated gene conversion rather than V(D)J recombination for antibody diversification. Recent gene knockout studies reveal paradoxical BCR signaling requirements for post-hatched chicken B cell development, with *J_H_* knockout chickens lacking post-hatched B cells, while recombination activating gene 1 (*RAG1*) knockout chickens maintain post-hatched bursal B cell populations through alternative pathways. Single-cell RNA sequencing has identified previously unrecognized chicken B cell subpopulations and provided molecular signatures for bursal and post-bursal B cells, addressing longstanding phenotypic marker limitations. These findings demonstrate that effective chicken humoral immunity can be achieved through alternative evolutionary strategies, with reduced dependence on RAG1 activity compared to mammalian systems, providing new perspectives on immune system evolution and adaptive immunity mechanisms.

## 1 Introduction

B lymphocytes are essential mediators of humoral immunity, responsible for generating protective antibodies through processes of receptor diversification, clonal selection, and differentiation into memory and plasma cells (1, 2). In mammals, B cell development occurs continuously throughout life in the bone marrow and follows a well-established sequence of recombination activating gene (RAG)-mediated V(D)J recombination, pre-B cell receptor (pre-BCR) signaling, and antigen-driven maturation in polarized germinal centers (3). This mammalian paradigm has long served as the foundation for our understanding of adaptive immunity and immunological memory.

However, accumulating evidence indicates that this model is not universally conserved across vertebrates. Among avian species, the chicken exhibits a strikingly different strategy for B cell development that is anatomically, temporally, and mechanistically distinct from that of mammals. Rather than relying on continuous lymphopoiesis in the bone marrow, chickens utilize a developmentally restricted program centered in the bursa of Fabricius—an avian-specific primary lymphoid organ—where B cells undergo rapid clonal expansion and diversification during embryonic and early post-hatch life (4). Notably, immunoglobulin diversity in chickens is not generated through extensive combinatorial V(D)J recombination but instead through activation-induced cytidine deaminase (AID)-mediated gene conversion using upstream pseudogenes (5–7). Moreover, recent *RAG1* and *J_H_* knockout models have revealed developmental stages in chickens that are partially independent of B cell receptor (BCR) signaling, challenging the long-standing assumption that RAG activity is universally required for early B cell development in vertebrates (8, 9). These observations suggest that adaptive immunity has evolved through multiple distinct strategies that converge on the shared objective of generating a functionally diverse antibody repertoire. The chicken immune system therefore provides a powerful comparative model to investigate fundamental principles of B cell biology, immune organ evolution, and somatic diversification mechanisms.

This review highlights unique features of chicken B cell development compared to humans. We specifically present recent findings on chicken B cell development, leveraging insights from newly established gene knockout chicken models and single-cell RNA sequencing (scRNA-seq) technology. Furthermore, we discuss current knowledge gaps and contradictory findings to emphasize the evolutionary divergence of avian B cell biology and redefine our understanding of vertebrate adaptive immunity for providing new perspectives for applications of chicken models in immunology, vaccine development, and antibody engineering.

## 2 Unique chicken B cell ontogeny compared to human

**Figure 1:**
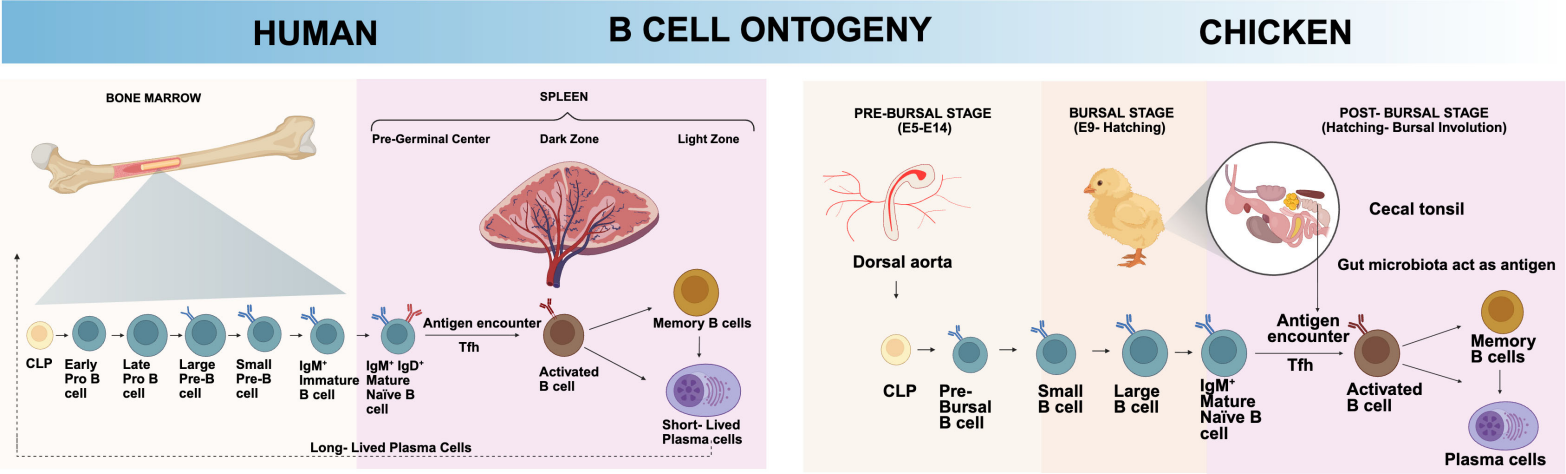
Unique chicken B cell ontogeny. Chicken B cells exhibit several unique developmental and functional features that set them apart from their human counterparts. The ontogeny of chicken B cells centers around the bursa of Fabricius, encompassing the pre-bursal, bursal, and post-bursal stages. In contrast, human B cell development primarily occurs within the bone marrow. Unlike the sequential RAG-mediated recombination of immunoglobulin genes in humans—where heavy chain rearrangement precedes light chain—chicken B cells undergo simultaneous recombination of both heavy and light chains. Created with BioRender.com.

The chicken B cell developmental program can be systematically divided into three distinct temporal and anatomical stages: pre-bursal, bursal, and post-bursal phases, each characterized by unique molecular signatures and functional requirements. This is contrasting with human B cell development program that follows a hierarchical progression from hematopoietic stem cells (HSCs) to mature immunocompetent B cells, occurring primarily in the bone marrow throughout life. HSCs first differentiate into multipotent progenitors, followed by lymphoid-primed multipotent progenitors, and common lymphoid progenitors (CLPs), which represents the first fully lymphoid-committed cells capable of generating lymphocytes, including B cells (10–12). From CLPs, the B cell-specific developmental pathway initiates with the generation of early pro-B cells, which then progress through late pro-B cells, large pre-B cells with VDJ of heavy chain rearranged first, small pre-B cells with VJ of light chain rearranged, and finally surface IgM (sIgM)^+^ immature B cells before emigrating from the bone marrow (3, 13). As chicken B cells undergo simultaneous rearrangement of immunoglobulin heavy and light chains, the conventional pre-B cell stage observed in mammals is absent (14). Consequently, the anatomical context of chicken B cell development, both within and outside the bursa of Fabricius, plays a critical role in shaping B cell ontogeny (**Figure 1**).

## 3 Pre-bursal stage B cell development

### 3.1 Origin of chicken B cell progenitors and lineage commitment

Chicken B cell development begins during early embryogenesis and is temporally restricted to a defined developmental window, in contrast to the continuous postnatal lymphopoiesis observed in mammals. The early cytoplasmic immunoglobulin expressing cells were detected in the chicken yolk sac around embryonic day (E)3 (15). However, primitive hematopoiesis (E1-E5) at the yolk sac primarily produces erythroid and myeloid lineages, but not lymphoid cells (16, 17). The definitive hematopoiesis (E2.5-E4 onward) generates multipotent HSCs capable of producing all blood lineages, including lymphoid cells (B and T cells). In chickens, this occurs at para-aortic foci and the dorsal aorta region, equivalent to the mammalian aorta gonad-mesonephros (AGM) within the intra-embryonic mesenchyme emerging at approximately E2.5-E3 (18, 19). These HSCs arise from hemogenic endothelium via an endothelial-to-hematopoietic transition (18, 20). B cell commitment occurs independently of the bursa of Fabricius, accompanied by simultaneous heavy and light chain V(D)J rearrangements at E7 in para-aortic foci and intra-embryonic mesenchyme (21). These V(D)J recombination is restricted to pre-bursal stage and not occurring in the bursal stage (22) These stem cells enter embryonic circulation and migrate to intermediate hematopoietic organs, including the spleen, and bone marrow, where early lymphoid progenitors transiently reside before homing to the bursa of Fabricius (23). Pre-bursal B cells are first detectable in the embryonic spleen at E10 (24). By E14, the embryonic spleen and bone marrow contain pre-bursal B cells that have undergone immunoglobulin rearrangements (25, 26).

Although the molecular mechanism of chicken pre-bursal B cells is less well characterized than early B lymphopoiesis in mouse or human bone marrow, several key pathways appear conserved (24, 27). In mammals, E2A serves as the earliest stage of B cell commitment, required for maintenance of the HSC pools, and are gradually increased from pre-pro B to pre-B stage for B cell commitment (28, 29). EBF1 and PAX5 synergistically regulate B cell lineage. EBF1 drives hallmark B cell gene expression (*CD19*, *CD79B*) by enabling PAX5 to interact with the MLL H3K4 methyltransferase complex, and it directly binds to the *PAX5* promoter to positively regulate its expression (30, 31). The activated PAX5 activates B cell specific genes and repress lineage-inappropriate genes, enforcing B cell fate (30, 32). IKAROS is a master regulator of lymphopoiesis, expressed throughout B cell development from lympho-myeloid primed progenitor (LMMP) to immature B cells (33). Low to moderate PU.1 level permits B cell development in concert with IRF8, while high PU.1 expression induces myeloid lineage commitment (34, 35). Additionally, critical signaling pathways for mammalian B cell development has been revealed. IL-7R signaling is essential for pro-B and pre-B cell proliferation, survival and differentiation via the JAK1/3-STAT5 pathway (36, 37). BAFF-BAFF-R signaling is essential for mature B cell survival via both canonical and non-canonical NF-κB pathways (38, 39). Wnt/β-catenin signaling influences B cell progenitor proliferation and survival, but excessive β-catenin activity blocks B cell differentiation (40, 41). Several direct evidences of transcription factors for chicken pre-bursal B cells have been reported. These involve PAX5 and EBF which are expressed in intraembryonic chicken hematopoietic precursors and embryonic spleen cells at E7-14, when pre-bursal stem cells are migrating to the bursa (42). The IKAROS transcription factor is also expressed in early chicken lymphoid progenitors that have T and B lymphoid potential before bursal or thymic colonization (43). However, pre-bursal chicken B cells in E10 spleens were BAFF-R negative, indicating BAFF-R expression begins during or after bursal colonization (24). Further studies are required to validate whether chicken pre-bursal B cell development is dependent on transcription factors or signaling pathways found in mammals.

Pre-bursal chicken B cells begin to express Bu-1 (also known as chB6) and IgM on their surface (25, 44). As V(D)J recombination occurs prior to bursal colonization, immunoglobulin lambda light chain is detected in E7 intraembryonic pre-bursal B cells (21). Pre-bursal B cell progenitors also express the carbohydrate structure sialyl Lewis x (CD15s), a selectin ligand that mediates adhesion to bursal vascular endothelium. Soon after migration into the bursa, bursal B cells undergo developmental switch and terminate sialyl Lewis x expression (45). With CD45, a pan-leukocyte marker, these markers can be used for tracing chicken pre-bursal B cells (46).

### 3.2 Restricted diversity through V(D)J recombination in pre-bursal B cells

**Figure 2:**
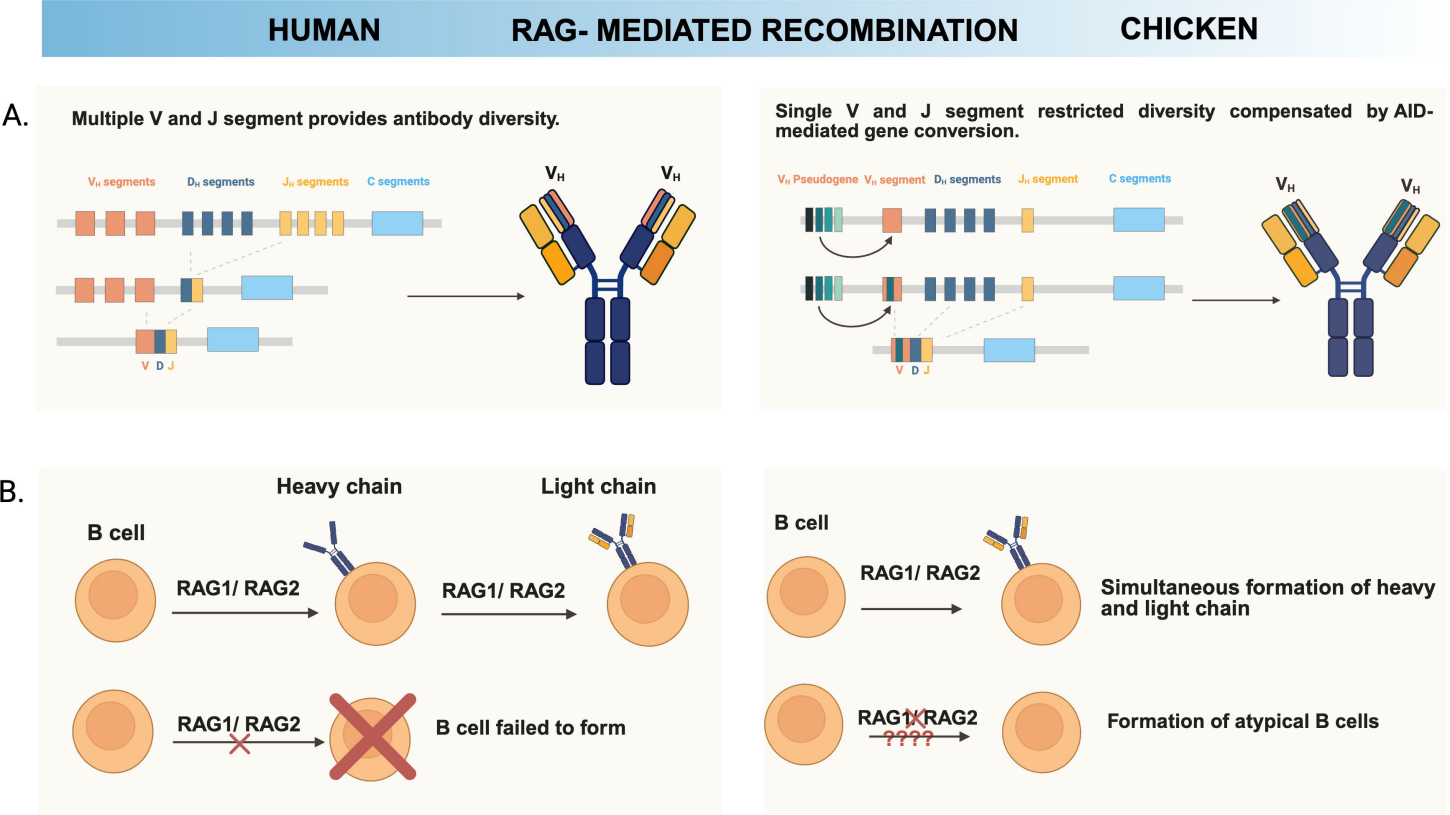
Chicken B cell development shows reduced dependency on RAG1 activity. A distinctive property of chicken B cell development is their reduced dependency on functional RAG-mediated recombination. **(A)** Since chickens possess only a single functional V and J segment in both heavy and light chains, RAG1-mediated V(D)J recombination generates limited antibody diversity. To compensate for this restriction, chickens primarily employ gene conversion, a mechanism by which pseudogene-derived V segments are templated into the rearranged V region, thereby generating the majority of antibody repertoire diversity **(B)** In humans, halting RAG recombinase activity completely abrogates B cell development. However, in chickens, minor subset of B cells is colonized in the bursa even when RAG activity is disrupted, and residual B cells remain detectable after hatching. Collectively, these observations underscore their reduced reliance on RAG1-mediated recombination for development. Created with BioRender.com.

The pre-bursal B cell progenitors have completed immunoglobulin heavy and light chain gene rearrangements. RAG-mediated V(D)J recombination in pre-bursal B cells represents a fundamental process for generating functional BCRs, through the mechanisms and outcomes differ dramatically from mammalian systems due to the unique organization of chicken immunoglobulin loci. The chicken immunoglobulin gene structure is remarkably simplified compared to mammalian counterparts, possessing only a single functional heavy chain variable (V_H_) gene, a single heavy chain joining (J_H_) gene, and 16 (D_H_) genes, with high homology (47). Additionally, D_H_ gene (*IGHD*) 1 and 14 were preferentially used across all antibody classes, while IgA in cecal tonsil prefers *IGHD* 15 with reduced *IGHD* 14 usage (5). Similarly, the light chain locus contains only a single functional variable (V_L_) gene and a single joining (J_L_) gene, creating an extremely constrained primarily repertoire. As a result, while humans rely on extensive germline V, D, and J gene libraries to create primary diversity, chickens have evolved a two-stage system where limited primary recombination is followed by extensive secondary diversification termed gene conversion (48, 49) (**Figure 2A**).

## 4 Bursal stage B cell development

The bursal phase represents the most distinctive aspect of chicken B cell development, beginning from E8-E14 when a highly selective population of pre-bursal stem cells migrates to colonize the developing bursa anlage through hatching (45, 50). The cellular dynamics of bursal colonization demonstrate extraordinary selectivity in terms of founding cell numbers. Each of the 10,000-12,000 lymphoid follicles that comprise the mature bursa is colonized by only 2–5 B cell precursors during this initial seeding phase. This extremely limited founding population subsequently undergoes massive clonal expansion within individual follicles, creating genetically homogenous populations within each follicle while maintain genetic diversity between follicles (27, 51).

### 4.1 Molecular mechanism of B cell migration to the bursa of Fabricius

The molecular mechanisms governing chicken B cell migration into and out of the bursa are primarily orchestrated by the *CXCR4*/*CXCL12* chemokine axis, which creates a highly regulated system for controlling B cell trafficking. *CXCL12* expression shows high abundance in the bursa anlage at E10, creating a strong chemotactic gradient that attracts *CXCR4*-expresing B cell precursors. *CXCR4* is detectable on early chicken B cell stages and increases progressively during bursal development, reaching peak levels during the phase of active colonization and expansion. Post hatch, a distinct subpopulation of B cells emerges with hallmarks of emigrating cells, characterized by significantly lower *CXCR4* expression levels, functionally necessary for B cells to leave the *CXCL12*-rich bursa environment and migrate to peripheral lymphoid organs (27). It is further evidenced by *in vivo* blockade of CXCR4 at the time of chicken B cell precursor immigration showed strong inhibition of follicle development (52). *CXCR4* is expressed on both granulocytes and chicken B cells, yet only the *CXCR4*-expressing B cells efficiently migrate into the bursal buds, whereas *CXCR4*-expressing granulocytes largely remain excluded (27). It suggests that CXCR4 alone is insufficient and that additional signals are required to mediate entry of chicken B cells into the bursa. In this regard, the *CXCL13/CXCR5* chemokine axis can potentially regulate the migration of chicken B cells into the bursa evidenced by that *CXCR5*-expressing bursal chicken B cells bind to recombinant CXCL13, which is expressed in bursa (53, 54). In early rabbit gut-associated lymphoid tissue (GALT), where antibody diversity is generated predominantly by somatic gene conversion and somatic hypermutation following relatively limited V(D)J recombination, CXCL13 produced by follicular stromal and follicular dendritic cell is implicated in recruiting *CXCR5*-expressing B cells into developing primary follicles (55). These results suggest potential role of CXCR5 in chicken pre-bursal B cells into *CXCL13*-expressing bursa.

### 4.2 Dispensable role of BCR during early bursal development

One of the most distinctive features of chicken B cell development compared to human is the dispensable role of the BCR during early bursal colonization and initial expansion phases (**Figure 2B**). Truncated sIgM receptor (Tμ), whose V_H_ and Cμ1 (first constant region of IgM heavy chain) domains were deleted, was introduced in developing chicken B cell precursors. The chicken B cells expressing Tμ, therefore, was not able to associate with light chain and consist of incomplete BCR harboring Cμ2 to Cμ4 of sIgM. The Tμ-expressing chicken B cells supported all the early stages of bursal colonization without sIg expression and endogenously rearranged VJ_L_ genes underwent gene conversion although it was not expressed on the cell surface (56, 57). Another study showed that chicken B cells expressing chimeric receptor that contains murine extracellular and transmembrane CD8α and CD8β with cytoplasmic chicken Igα and Igβ for signal transduction supported all early stages of bursal B cell development (58). Recently, it was reported that CRISPR/Cas9-mediated *RAG1* knockout chicken B cells can develop and colonize the bursa in the absence of V(D)J recombination during chicken development, whereas BCR-mediated signaling becomes progressively more critical during later developmental stages (8). Additionally, light chain (Ig_L_) knockout chickens retain a small population of B cells in the bursa that can migrate to the spleen and blood, whereas heavy chain (J_H_) knockout chickens allow B cell entry into the bursa but completely lack mature peripheral B cells. This indicates that expression of immunoglobulin heavy chain alone, rather than light chain, is sufficient to support chicken B cell development (59). These findings collectively suggest that BCR-mediated signaling is dispensable for embryonic chicken B cell development until bursal phase.

### 4.3 Bursal B cell developmental progression

Bursal B cells exhibit a complex and dynamic pattern of developmental stage and functional state. The bursa is compartmentalized into cortex and medulla at late embryonic stage. Cortical B cells represent the actively diversifying population characterized by rapid proliferation, and gene conversion (60, 61). Cortical B cells also upregulate MHC class II molecules (60, 62) and maintain high *CXCL12* expression, creating a retention signal for proliferating B cells via *CXCR4* signaling (27). In contrast, medullary B cells show slow division rates, with limited gene conversion activity compared to cortical counterparts (60, 63). The medulla serves as an antigen-sampling interface connecting the bursal lumen to lymphoid tissue. The medulla also contains bursal secretory dendritic cells that may participate in antigen presentation and microenvironmental regulation (27, 64). The medullary B cells migrate into the cortex, and B cells survived from the diversification process migrate from cortex to periphery, seeding secondary lymphoid organs with mature naïve B cells.

The developmental progression of bursal B cells can be tracked through changes in cell size. During late embryonic development, particularly E17-E18, the bursal B cell population undergoes substantial expansion and segregates into two morphologically and functionally distinct subpopulations: small B cells and large B cells. Large B cells demonstrate superior proliferative capacity with higher expression of genes related to their survival such as *BAFF-R* compared to small B cells. Additionally, they expressed higher level of differentiation-associated genes such as *RAG1, FOXO1, FOXO3, PAX5*, and Ikaros family transcription factors (60). This indicates that large bursal B cells resemble with cortical B cells, while small bursal B cells resemble with medullary B cells.

### 4.4 Antibody diversification through gene conversion

The bursal environment triggers the most distinctive aspect of avian antibody diversification through an extensive gene conversion process that begins around E15 and fundamentally transforms the limited primary B cell repertoire (65, 66). Gene conversion is a non-reciprocal recombinational process where sequences from upstream pseudogenes (25 light chain pseudogenes, and approximately 80 or more heavy chain pseudogenes) replace homologous sequences in the functional, rearranged immunoglobulin V gene segments to compensate restricted diversity of chicken immunoglobulin (67, 68). The molecular mechanism of gene conversion involves AID, which initiates the process by deaminating cytidine nucleotides within the functional immunoglobulin gene, creating uracil residues that are recognized as DNA damage. Subsequent processing by the DNA repair machinery creates double-strand breaks that are repaired using homologous sequences from the upstream pseudogenes as templates (69). Several other factors were found to be involved in gene conversion process. Three translesion synthesis (TLS) polymerase genes (*POLH, POLN, POLQ*) knockout DT40 cell line completely abolished gene conversion and reduced somatic hypermutation (SHM), demonstrating that these error-prone polymerases perform the DNA synthesis step during homologous repair (HR)-mediated gene conversion (70). RAD51 paralogs and HR machinery defects shift AID-initiated lesions away from templated gene conversion toward mutational outcomes, indicating that HR factors are required to execute gene conversion rather than hypermutation (71, 72). Uracil DNA glycosylase (UNG) and mismatch/base excision repair components process AID-induced uracils and induce lesions repaired by templated gene conversion (71, 72). Additionally, chromatin structure and epigenetic regulation tunes gene conversion frequency. Various epigenetic repressors of gene conversion, such as histone deacetylase (HDAC), heterochromatin factor HP1, block epigenetic activation by reducing histone acetylation of antibody gene or pseudogene region, while TET3 mediates DNA demethylation of pseudogenes, causing elevated gene conversion (71, 73–76).

Unlike human B cells, where non-productive immunoglobulin gene rearrangements typically result in cell death or allelic exclusion, chicken B cells containing non-functional rearrangements can continue to undergo gene conversion within the embryonic bursa (14). Notably, non-functional sequences in humans accumulate somatic mutations (77), but this occurs in a minority of cells and does not lead to productive antibodies, whereas in chickens, gene conversion can restore functionality or generate diversity even from initially non-productive sequences (78, 79).

Chicken and mammalian antibody diversification diverge at both the germline and repertoire levels. At the germline level, chicken pseudogenes have fewer amino acid positions showing higher diversity compared to human germline functional genes. At the repertoire level, chickens maintain lower amino acid diversity compared to humans, especially in frame work regions. Heavy chain complementarity-determining region (CDR) 3 in chickens shows significantly less amino acid diversity per position compared to humans across all antibody classes. The length of chicken heavy chain CDR3 is larger than that of human, and chicken CDR3s are more hydrophilic than human CDR3s in heavy chains. It suggests that larger CDR3 loops provide greater structural flexibility in the antigen-binding site and higher hydrophilicity increase protein binding potential, making chicken antibodies more flexible and polyreactive (80). Another study showed that chickens exhibit strong biases in pseudogenes donor usage during gene conversion. At the heavy chain locus, 8 of more than 80 V_H_ pseudogenes were consistently preferred across all birds, with 14 more pseudogenes preferred in at least one bird; at the light chain locus, 8 of 25 V_L_ pseudogenes were generally preferred, with 15 additional pseudogenes preferentially used in one or more birds (5). The resultant diversified chicken immunoglobulin exhibits diverse binding topologies distinct from mammalian antibody structure. Chicken single chain fragment variable (scFv) antibodies exhibit intra-CDRH3 disulfide bond (e.g., Cys-94-Cys-102 forming a rigid 14 residue heavy chain CDR3) that structurally pairs with a unique light chain CDRL1. This combined topology is absent in mammalian antibodies and are prevalent (>50%) of the chicken antibody repertoire due to gene-conversion-based immunoglobulin diversification (81). These structural attributes enable chickens to generate unconventional paratopes capable of binding epitopes poorly recognized by mammalian antibodies (81, 82).

### 4.5 Negative and positive selection of bursal B cells

Within the bursal follicles, B cells explosively proliferate, but 90-95% of newly generated bursal B cells die by apoptosis, and only a small fraction emigrate to peripheral lymphoid tissues. It indicates that robust negative selection is occurring in the bursal follicle (83), When chimeric receptors composed of lamprey variable lymphocyte receptor (VLR) diversity regions specific to HEL (self-antigen hen egg lysozyme) fused with chicken IgM constant region (Tμ) were expressed in developing chicken B cells, it resulted in complete deletion of HEL-recognizing B cells *in vivo*. Additionally, E13 pre-bursal B cells expressing chimeric Tμ fused with lamprey VLR specific to phycoerythrin (PE) (VLR^PE^Tμ-expressing B cells) are deleted when soluble PE is *in ovo* intravenously given. It was also found that when the chicken Igα signaling motifs of BCR was disrupted, B cells expressing the surrogate receptor were not deleted, even in the presence of ligands. These results suggest that chicken B cells are subject to clonal deletion when their BCR is self-reactive even outside of the bursa, and BCR signaling is indispensable for negative selection (84).

Following diversification and proliferation in bursal follicles, only a small percentage of cells emigrate to the periphery B cells. Only B cells with productive, non-self-reactive BCRs that can receive appropriate survival signals are positively selected to move from medulla to cortex, and subsequently emigrate as peripheral B cells (61, 83). Bursal microenvironmental cues are essential for guiding B cell migration and sustaining their survival. BAFF is a key trophic factor for immature and post-hatch bursal B cells (24), while downregulation of *CXCR4* expression is required for their emigration from the bursa to peripheral lymphoid organs (27). After hatch, B cell persistence requires surface expression of an intact variable (V) region on the BCR. In contrast, chicken B cells, expressing Tμ or CD8α:Igα chimeric receptors, both lacking a V region, failed to maintain B-cell development after hatch (85, 86). Schusser et al. demonstrated that while B cell precursors can colonize the bursa without functional immunoglobulin expression, they cannot survive in the peripheral compartment. In *J_H_* knockout chickens, absence of heavy chain led to failure of B cell survival, and bursal follicles were devoid of B cells post hatch. As a result, post-bursal B cells found in peripheral organ were absent in the *J_H_* knockout chicken (9). Recent study using *RAG1*-deficient chickens demonstrated that impaired BCR leads to insufficient B cell activation responses, decreased expression of *AID*, and impaired ability to undergo further antibody diversification in response to antigenic stimulation although small number of bursal B cells existed after hatch. These results indicate that BCR dependency increases during chicken B cell development, particularly in the post-hatch period (8).

Two main models have been proposed to explain how BCR operates for positive selection after hatch. One model suggests that gut-derived bacterial superantigens bind BCRs in a largely specificity-independent manner, delivering survival signals to many bursal B cells regardless of their individual antigen specificity. In contrast, a second model suggests that only those B cells whose BCRs acquire appropriate specificity for gut-derived antigens receive sufficient signals to support cortico–medullary redistribution and long-term maintenance after hatch (83). Indeed, B cells engineered to express a chimeric CD8α:Igα receptor are stably maintained *in vivo* when anti-CD8α antibodies are administered intrabursally, demonstrating that surrogate ligation of this receptor can substitute for antigen-specific BCR engagement (86). Likewise, VLR^PE^Tμ-expressing B cells can migrate to the periphery without antigen by E18, but their numbers in both bursa and periphery declined rapidly after hatch if antigen is absent. Intravenous PE injection at the pre-bursal stage (E13) deleted pre-bursal VLR^PE^Tμ-expressing B cells, with PE behaving as a self-antigen that induces negative selection (84). However, intrabursal PE injection leads to selective survival of bursal and peripheral VLR^PE^Tμ-expressing B cells after hatch, with PE acting as a foreign antigen that drives positive selection (87). Together, these results indicate that engagement of surface immunoglobulin can mediate both negative selection of pre-bursal B cells recognizing self-antigens and positive selection of bursal B cells recognizing foreign gut-derived antigens.

## 5 Post-bursal stage B cell development

Following hatching, chicken B cells that have completed clonal expansion and immunoglobulin diversification within the bursa of Fabricius begin to emigrate to peripheral lymphoid organs, including the spleen, cecal tonsils, and gut-associated lymphoid tissues until bursal involution (88, 89). This post-bursal stage is characterized by a transition from a developmentally programmed, antigen-independent phase to an antigen-driven phase of B cell maturation and functional activation, which parallels but also diverges in key aspects from human postnatal B cell development.

### 5.1 Differential gene expression of post-bursal B cells

Post-bursal B cells exhibit distinct phenotypic characteristics that distinguish them from their bursal precursors and reflect their functional maturation. Post-bursal B cells show altered expression patterns of chemokine receptors, particularly the reduced *CXCR4* expression that enables their exit from the *CXCL12*-rich bursal environment (27). The *BAFF-R*, which is highly expressed on bursal B cells, shows progressive downregulation during differentiation toward plasm cell lineages, while its expression is upregulated during human B cell development from the immature to transitional stages (90). This downregulation correlates with activation and differentiation status, suggesting that *BAFF-R* expression level can serve as markers for chicken B cell maturation stage and functional capacity. For *Bu-1*, most peripheral chicken B cells maintain *Bu-1* expression, but plasma cells show dramatic reduction or complete loss of *Bu-1* expression, providing a marker for terminal B cell differentiation (91).

### 5.2 Unique features of chicken antibody class switch recombination

Post-bursal B cells undergo species-specific class switch recombination (CSR), the process which modifies IgM constant region into other isotype antibodies, while keeping antigen specificity (92). Both humans and chickens require AID for initiating CSR. AID introduces U:G mismatches in repetitive switch (S) region DNA upstream of heavy chain constant genes, which leads to DNA double strand breaks after processing by base excision repair enzyme UNG (93). In humans and mice, UNG deficiency greatly impairs CSR, though some residual switching still occurs via the alternative mismatch repair (MMR) pathway (94, 95). The MSH2/MSH6 heterodimer recognizes AID-induced U:G mismatches and, through coordinated action with EXO1 and error-prone polymerases, generates overlapping gaps that resolve into double-strand breaks required for CSR. Only combined UNG–MSH2 or UNG–MSH6 deficiency completely abolishes CSR (reducing switching to <1–2% of wildtype), underscoring the parallel and non-redundant roles of these pathways in processing AID-induced lesions (96–98). Chickens possess orthologous UNG and MMR proteins, and studies in the DT40 chicken B-cell system indicate that both pathways contribute to processing AID-induced lesions during immunoglobulin diversification (99). The DNA double-strand breaks in switch regions are ultimately resolved by non-homologous end joining (NHEJ) in both species. The fundamental machinery is shared – for example, chickens have identifiable Sμ and Sυ (IgY) switch regions, and recombination between Sμ and Sυ deletes the Cμ gene much as Sμ–Sγ recombination does in humans (100).

The regulatory signals governing CSR are likewise broadly shared. CD40–CD40L interactions between T helper cells and B cells are essential in both humans and chickens, activating NF-κB signaling that induces AID expression and B-cell differentiation in chickens (101–103). Notably, NF-κB family transcription factors such as p50 and c-Rel facilitate AID targeting to Ig genes in chicken B cells (101), paralleling their critical role in human CSR downstream of CD40 engagement. Cytokine signals then direct the specific isotype outcome. IL-10 enhances class switching in both species where IL-10 synergizes with CD40L to induce IgG in human B cells (104), and in chickens IL-10 plus CD40L robustly induces switching to IgY and IgA *in vitro* (91). IL-21, produced by Tfh cells, influences CSR and plasma cell differentiation across vertebrates promoting IgG or IgA production while curbing IgE in humans (105–107). Chickens express functional IL-21 predominantly in CD4^+^ TCRαβ^+^ T cells, and chicken IL-21 (chIL-21) acts as a T cell costimulatory cytokine that synergistically enhances T cell proliferation (108). Given the conserved structure of IL-21 between mammals and chickens and the presence of IL-21-responsive T-B cell interactions in chickens, it is likely that chicken IL-21 similarly influences class switching to IgY and IgA, though direct experimental evidence remains to be established. These cytokine-driven signals converge on transcriptional regulators that orchestrate CSR.

Despite these mechanistic similarities, there are notable species-specific differences in CSR outcomes and context. Humans can produce five heavy-chain isotype which are IgM (μ), IgD (δ), IgG (γ), IgA (α), and IgE (ϵ), whereas chickens contain three functional isotype which are IgM, IgY (υ, equivalent to human IgG), and IgA, arranged in a unique μ-α-υ configuration that differs from the human μ-δ-γ-ϵ-α arrangement (109, 110). Another evolutionary adaptation in chicken physiology is the presence of IgY as the major systemic antibody isotype. Chicken IgY serves roles equivalent to human IgG, including systemic immune protection and maternal antibody transfer to offspring through egg yolk (100). Structurally, avian IgY heavy chains have four constant domains and no hinge region resembling IgE, whereas IgG has three constant domains and a hinge (111). Mammals evolved separate IgG and IgE classes to specialize these functions, but birds rely on a single IgY class to fulfill both roles (112–114). Additionally, the molecular organization of the chicken heavy chain constant region locus shows remarkable compactness compared to human counterparts. The entire locus spans only 67 kilobases, significantly shorter than human immunoglobulin heavy chain loci. There are no introns between the CH1 and CH2 domains of the IgY. Another unique feature is that IgA gene is inverted relative to the IgM and IgY genes, showing transcriptional orientation opposite to the other constant region genes (109). Overall, avian class switching yields a more limited repertoire of antibody isotypes than human CSR, which correlates with differences in immune system organization.

### 5.3 Peripheral diversification and germinal center reaction

**Figure 3:**
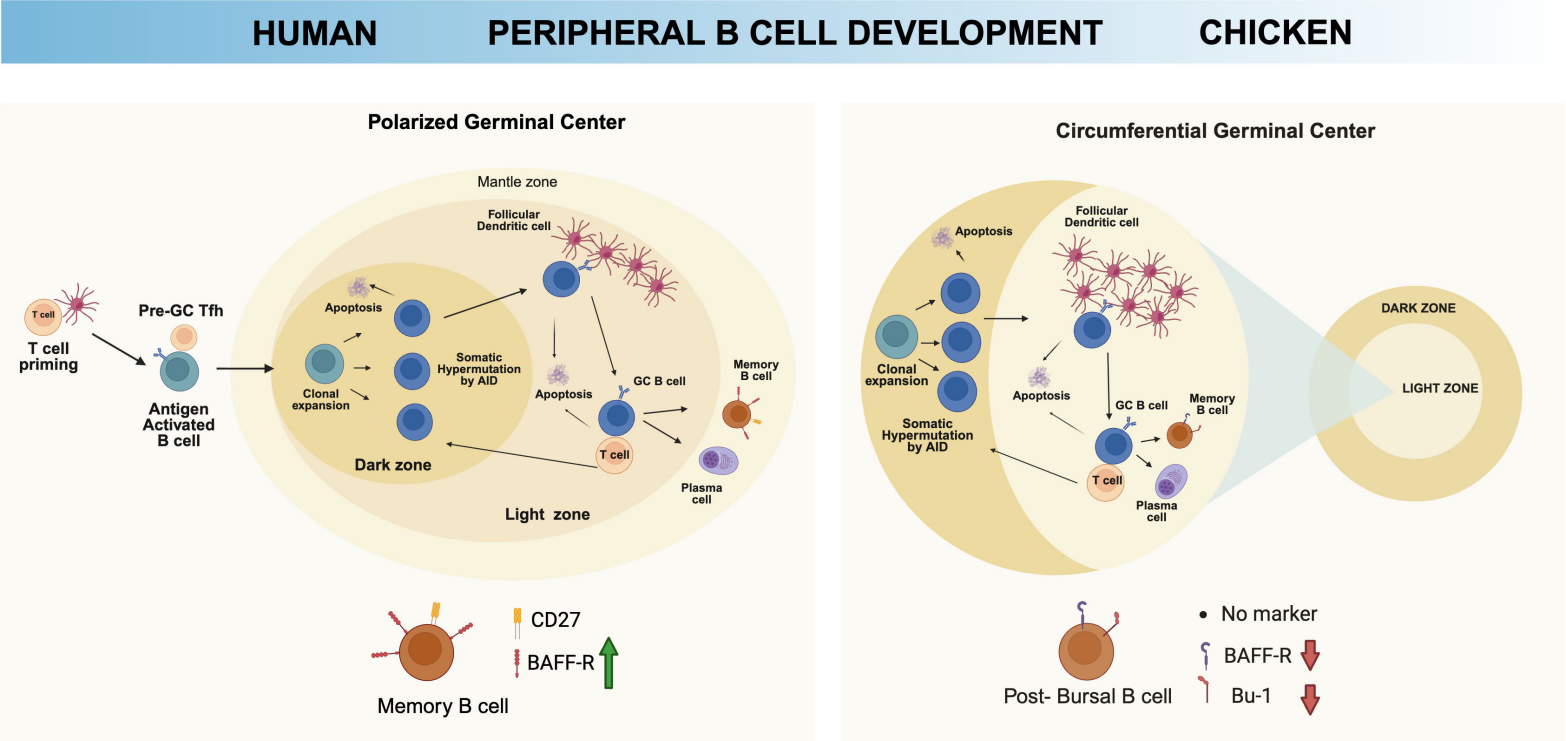
Distinct feature of peripheral chicken B cell development. Upon migration to peripheral tissues, antigen-driven B cell maturation occurs in the germinal center (GC). While humans exhibit polarized GC organization, chickens display a circumferential GC structure for B cell activation and maturation. Furthermore, *BAFF-R* expression is gradually upregulated until transitional B cells in humans, while its expression keeps downregulated in chicken post-bursal B cells. Created with BioRender.com.

Upon encountering environmental antigens, post-bursal B cells participate in peripheral germinal center (GC) reactions within the secondary lymphoid tissues such as spleen, cecal tonsils and bronchus-associated lymphoid tissue (BALT) (115, 116). Unlike mammals, which exhibit polarized dark and light zone architecture (115, 117), chicken GCs display a distinctive circumferential organization, where proliferating centroblasts are located at the periphery and selection with the help of dendritic cells capable of trapping immune complexes occurs in the central region (118) (**Figure 3**). Surrounding the splenic vasculature, the white pulp consists of the T cell-rich periarterial lymphatic sheath (PALS) encircling the central artery. The artery divides into penicillary capillaries wrapped in the periellipsoid lymphocyte sheath (PELS), where ellipsoids are embedded. Connective tissue-encapsulated GCs typically form at the arterial branch points (119, 120).

Antigen-activated chicken B cells in the periphery differentiate into memory B cells and plasma cells, similar to mammalian systems, but with avian-specific regulatory features. Memory B cells in chickens have been potentially identified functionally rather than phenotypically due to the lack of conserved surface markers such as CD27 (121). Class-switched chicken B cells declined *BAFF-R* expression when stimulated with CD40L/IL-10 (91), contrasting with human systems where memory B cells typically remain *BAFF-R* expression although *BAFF-R* deletion in murine model showed that memory B cells can survive without BAFF-R signaling (122–124). When terminally differentiated into plasma cells, *BAFF-R* expression becomes lost (91, 125). Evidence shows that chickens, like mammals, possess long-lived plasma cells (LLPCs) that can maintain serum antibody levels independent of continued antigen stimulation. These cells have been detected in chicken spleens up to 18 weeks post-infection, which differs from mammalian LLPCs residing in bone marrow niches (121).

## 6 Evolutionarily and mechanistic insights from chicken B cell development

### 6.1 Critical gaps and emerging perspectives in chicken B cell development research

Despite decades of research establishing chickens as a foundational model for B cell biology, significant gaps remain in our understanding of avian lymphocyte development that limit both basic research applications and translational potential. The major limitations include the absence of reliable phenotypic markers for chicken B cell subsets, paradoxical BCR signaling requirements revealed through genetic knockout studies, and the unexplored implications of RAG1’s diminished role in avian versus mammalian B cell development. Recent advances in single-cell technologies and CRISPR-mediated genetic chicken models now provide unprecedented opportunities to address these fundamental knowledge gaps.

### 6.2 Single-cell RNA sequencing reveals hidden chicken B cell diversity

Chicken B cell research has been severely constrained by the absence of species-specific markers for distinguishing multiple B cell subsets. Traditional reliance on *Bu-1* as a pan-chicken B cell marker, combined with morphological criteria, has proven insufficient for reliable subset identification (91). While mammalian system utilizes well-established markers like *CD27* for memory cells and *CD138* for plasma cells, CD27 expression has not been reported on chicken B cells, and CD138 is only transiently upregulated on chicken B cells activated in culture with CD40L and IL-10, rather than serving as a plasma cell marker (91). scRNA-seq technology can fill in the gap of marker absence. Recent scRNA-seq in post-hatch bursa identified 16 B cell clusters, and further subdivided them into large and small subsets. Large B cells expressed higher level of classical B cell differentiation and signaling genes than small B cells. The microbiota depletion perturbs normal B cell maturation dynamics even when total *Bu-1*-expressing B cell frequency appears similar at later time points. This indicates that the gut microbiota is a critical regulator of bursal B cell development in young broiler chickens. Authors also discovered a previously uncharacterized B cell subpopulation and identified *Taf1* as a novel transcriptional regulator associated with B cell lineage differentiation in the bursa (126). scRNA-seq in chicken peripheral blood identified 31 distinct leukocyte clusters including multiple previously unrecognized B cell subpopulations. The chicken B cell clusters were identified by the expression of *Bu-1*, *CD79A*, *CD79B*, *PAX5*, and several immunoglobulin related genes such as *IGLL1*, *VH26L1*. Most significantly, a novel chicken B cell subset characterized by high *SOX5* expression was discovered (127). Another scRNA-seq study on chicken spleen infected with Marek’s disease virus identified antigen-presenting B cells, plasma cells. Especially, *IGLL1*, *JCHAIN*, and *TXNDC5* were used for annotating chicken plasma cells (128). In mammals, XBP1, regulated by PRDM1 supports immunoglobulin synthesis plasma cells, thus acting as plasma cell markers (129, 130). Chicken *in vitro* B cell differentiation study using CD40L and IL-10 showed that PRDM1 expression patterns align with plasma cell differentiation programs, providing molecular foundations for subset discrimination (91). It remains necessary to validate whether plasma cell markers such as *PRDM1* and *XBP1* can reliably distinguish chicken plasma cells from other B cell subsets at single-cell resolution. The development of antibodies specific for each chicken B cell subset identified through scRNA-seq will be critical to ensure precise and robust cellular analysis.

### 6.3 BCR signaling paradox for post-hatched chicken B cells revealed through gene knockout chicken models

Recent gene knockout studies revealed paradoxical BCR signaling requirements that challenge current models of chicken B cell development. Traditionally, embryonic chicken B cell can develop without BCR component, but it becomes indispensable for B cell development post-hatch (56–58, 85, 86). *J_H_* knockout chicken model supported this model by demonstrating that membrane heavy chain/BCR expression is dispensable for bursal colonization, proliferation, and gene conversion, as these processes proceed normally despite complete BCR absence. However, the loss of post-hatching chicken B cells indicates the BCR signaling becomes absolutely essential for peripheral survival and emigration (9). These features contrast with B cell development in rabbit which is another species using GALT for B cell development. Rabbit B cell diversification requires antigen stimulation from gut microbiota in GALT, whereas chicken B cell diversification occurs in the sterile embryonic bursa independent of exogenous antigens (131–133). The *RAG1*- and *RAG2*-knockout rabbit models resulted in absence of organized appendix/IPP GALT and of B cell precursor development, while BCR-independent chicken B cells can develop in the embryonic bursa (57, 134). Together, these findings reveal that chicken B cell ontogeny operates under uniquely stage−restricted and organ−specific BCR requirements that differ fundamentally from the strictly BCR− and microbiota−dependent GALT pathway in rabbits.

The *RAG1* knockout chicken model fundamentally challenges established paradigms of chicken B cell development by revealing critical distinctions between complete immunoglobulin absence and incomplete V(D)J recombination. *RAG1*-deficient chickens retain IgM^+^ bursal B cells throughout development, from embryonic stages to 3 weeks post-hatching, contrasting sharply with *J_H_* knockout chickens that exhibit complete bursal B cell absence after hatching despite normal embryonic colonization. This phenotypic divergence stems from distinct molecular mechanisms: *RAG1*-deficient chicken B cells can express truncated immunoglobulins containing only J and C segments through alternative recombination or transcriptional mechanisms, whereas *J_H_* knockout chickens completely lack the J_H_ segment required for any heavy chain expression (8, 9). These findings suggest that minimal immunoglobulin expression-even without functional V(D)J recombination-can support bursal B cell survival independent of RAG1 activity. However, the functional significance of this alternative developmental pathway requires validation through examination of peripheral B cell populations in post-hatched *RAG1* knockout chickens.

### 6.4 RAG1’s diminished role in chicken B cell development compared to mammals

Human *RAG1* deficiency presents a diverse clinical spectrum depending on the pattern of the *RAG1* disruption. Complete loss-of-function mutations in human *RAG1* cause severe combined immunodeficiency with complete absence of mature T and B cells (135). In contrast, hypomorphic human *RAG1* mutations with residual recombinase activity frequently lead to combined immunodeficiency with granulomas and autoimmunity (136). Recent studies demonstrate that partial human *RAG* deficiency creates a unique immunological environment characterized by restricted primary BCR repertoires enriched for autoreactive specificities (137). The lymphopenic environment in these patients elevates serum BAFF levels, allowing autoreactive transitional B cells that would normally eliminate to survive and undergo homeostatic proliferation (138). This process generates double-negative (IgD^-^CD27^-^) B cells and memory B cells that can rapidly differentiate into IgG-secreting plasma cells upon stimulation, leading to elevated autoantibody production.

However, complete *RAG1* deficiency in chickens produces different developmental outcomes that challenge mammalian-centric paradigms of B cell biology. Unlike humans where *RAG1* loss prevents B cell development entirely, *RAG1*^^-^/^-^^ chickens successfully develop and maintain small number of bursal B cell populations post-hatching despite complete absence of RAG1 activity. However, *RAG1*-deficient B cells showed impaired gene conversion with attenuated BCR signaling (8). These molecular defects suggest that *RAG1*-deificent B cells, while phenotypically present, may develop through alternative pathways, potentially generating populations with atypical functional characteristics including enhanced autoreactive potential. Given the established association between partial *RAG1* deficiency and autoimmunity in humans (137), comprehensive functional characterization of *RAG1-*deficient chicken B cells is needed to determine whether the apparent developmental rescue masks underlying autoimmune predisposition similar to human hypomorphic RAG1 syndromes.

## 7 Conclusion

The comparative analysis of chicken B cell development reveals a remarkable evolutionary divergence from mammalian paradigms, characterized by unique three-stage ontogeny, restricted RAG-mediated recombination, and distinct structure of peripheral GC structure. While chickens achieve effective humoral protection through alternative mechanisms, including AID-mediated gene conversion, BCR-independent early development, and circumferential GC architecture, critical knowledge gaps persist, particularly regarding phenotypic markers for chicken B cell subsets, the molecular basis of contradictory knockout phenotypes (*J_H_*^-/-^ versus *RAG1*^-/-^), and the diminished role of chicken RAG1 in B cell development compared to mammals. Recent advances in scRNA-seq and CRISPR-mediated genetic chicken models provide unprecedented opportunities to address these limitations, potentially revealing novel regulatory mechanisms that support robust immune function through pathways distinct from mammalian systems. Understanding these species-specific adaptations not only advances fundamental immunology but also enhances the translational potential of chicken models for biomedical research, vaccine development, and agricultural applications. Furthermore, it will demonstrate multiple evolutionary strategies of avian B cell maturation beyond the traditional mammalian paradigm.
